# Maternal Glucose and LDL-Cholesterol Levels Are Related to Placental Leptin Gene Methylation, and, Together With Nutritional Factors, Largely Explain a Higher Methylation Level Among Ethnic South Asians

**DOI:** 10.3389/fendo.2021.809916

**Published:** 2021-12-24

**Authors:** Line Sletner, Aina E. F. Moen, Chittaranjan S. Yajnik, Nadezhda Lekanova, Christine Sommer, Kåre I. Birkeland, Anne K. Jenum, Yvonne Böttcher

**Affiliations:** ^1^ Department of Pediatric and Adolescents Medicine, Akershus University Hospital, Lørenskog, Norway; ^2^ Institute of Clinical Medicine, University of Oslo, Lørenskog, Norway; ^3^ Department of Clinical Molecular Biology, Akershus University Hospital, Lørenskog, Norway; ^4^ Division of Infection Control and Environmental Health, The Norwegian Institute of Public Health, Oslo, Norway; ^5^ Diabetes Unit, King Edward Memorial (KEM) Hospital and Research Centre, Pune, India; ^6^ Department of Endocrinology, Morbid Obesity and Preventive Medicine, Oslo University Hospital, Oslo, Norway; ^7^ Institute of Clinical Medicine, University of Oslo, Oslo, Norway; ^8^ General Practice Research Unit, Department of General Practice, Institute of Health and Society, Faculty of Medicine, University of Oslo, Oslo, Norway

**Keywords:** Leptin, placenta, methylation, cholesterol, ethnicity, gestational diabetes

## Abstract

**Background:**

Leptin, mainly secreted by fat cells, plays a core role in the regulation of appetite and body weight, and has been proposed as a mediator of metabolic programming. During pregnancy leptin is also secreted by the placenta, as well as being a key regulatory cytokine for the development, homeostatic regulation and nutrient transport within the placenta. South Asians have a high burden of type 2 diabetes, partly attributed to a “thin-fat-phenotype”.

**Objective:**

Our aim was to investigate how maternal ethnicity, adiposity and glucose- and lipid/cholesterol levels in pregnancy are related to placental leptin gene (*LEP*) DNA methylation.

**Methods:**

We performed DNA methylation analyses of 13 placental *LEP* CpG sites in 40 ethnic Europeans and 40 ethnic South Asians participating in the STORK-Groruddalen cohort.

**Results:**

South Asian ethnicity and gestational diabetes (GDM) were associated with higher placental *LEP* methylation. The largest ethnic difference was found for CpG11 [5.8% (95% CI: 2.4, 9.2), p<0.001], and the strongest associations with GDM was seen for CpG5 [5.2% (1.4, 9.0), p=0.008]. Higher maternal LDL-cholesterol was associated with lower placental *LEP* methylation, in particular for CpG11 [-3.6% (-5.5, -1.4) per one mmol/L increase in LDL, p<0.001]. After adjustments, including for nutritional factors involved in the one-carbon-metabolism cycle (vitamin D, B12 and folate levels), ethnic differences in placental *LEP* methylation were strongly attenuated, while associations with glucose and LDL-cholesterol persisted.

**Conclusions:**

Maternal glucose and lipid metabolism is related to placental *LEP* methylation, whilst metabolic and nutritional factors largely explain a higher methylation level among ethnic South Asians.

## Background

During normal pregnancy, physiological changes in maternal body composition and glucose and lipid metabolism occur to ensure appropriate supply of nutrients to the growing fetus (1). After an initial small decrease in insulin resistance, fasting glucose and lipid levels in early pregnancy, there is a progressive increase in insulin resistance, triglycerides, high-density lipoprotein (HDL) cholesterol and low-density lipoprotein (LDL) cholesterol as pregnancy progress (2). Glucose is the major nutrient required for fetal growth and is primarily sourced from the maternal circulation and transported across placenta by facilitated diffusion (1). Triglycerides are hydrolysed by lipases on the maternal side of the placental syncytiotrophoblast, and free fatty acids are released and taken up by the placenta. Cholesterol is important for placental and fetal growth and maturation and necessary for steroid hormone synthesis, including estrogen. Cholesterol is probably mainly delivered to the placenta by LDL-cholesterol, taken up by endocytosis (3).

Leptin is a peptide hormone central for energy homeostasis. It is secreted mainly by the adipose tissue, regulating energy balance by inhibiting hunger (4). During pregnancy leptin is also secreted from the placenta, and the maternal circulating leptin levels increase substantially with increasing gestation (5, 6). Within the placenta leptin has physiological effects on placenta development including angiogenesis, growth and immunomodulation, and there is support for it’s role in the regulation of placental nutrient transport (e.g. glucose and amino-acids), by up-regulating specific placental nutrient transporter isoforms (5, 7). Expression of the leptin gene (*LEP)* is regulated in parts by epigenetic mechanisms. Several CpG islands are found in the *LEP* promoter region and methylation in these regions will affect the *LEP* expression (7–11).

Across the life span, ethnic South Asians have a high burden of type 2 diabetes and are diagnosed at a younger age and at a lower BMI than ethnic Europeans (12, 13). The increased risk is thought to be partly explained by South Asians being smaller and thinner at birth, but with relatively more adiposity, a phenotype that persists throughout life (14). This “thin-fat-phenotype” has been partly related to “the double burden of malnutrition”, defined as the simultaneous manifestation of both undernutrition (primarily micronutrient deficiencies) and overnutrition (increased adiposity, hyperlipidemia and hyperglycemia), also during pregnancy (15). It is likely that the placenta plays an important role for these relationships.

Studies of maternal obesity and diabetes have reported inconsistent findings of methylation or gene expression in placental tissue (16, 17). Most studies exploring epigenetic mechanisms in placenta have been performed in Caucasian populations, and some suggest that maternal obesity and hyperglycemia are associated with hyper-methylation and hypo-expression of the *LEP* gene in placenta (8, 10, 17). Few studies have examined relations with maternal lipid concentrations or micronutrient levels. Leptin has been proposed as a mediator of metabolic programming. Given the differences in body composition, as well as different exposures to socioeconomic and nutritional factors across the life course, studying relations between maternal adiposity, glucose and lipid metabolism and placental *LEP* methylation in ethnic South Asian and European mothers living in Europe could thus shed light on mechanisms involved in developmental programming of metabolic diseases.

We therefore investigated the relationships of maternal ethnicity (European vs. South Asian), adiposity, hyperglycemia and lipid levels with placental DNA methylation in *LEP*, also taking nutritional factors into account, in a well characterized Norwegian cohort of pregnant women.

## Materials and Methods

### Population and Design

Data are from a population-based cohort study of 823 presumably healthy pregnant women attending the Child Health Clinics in three city districts in Groruddalen, Oslo, Norway, for primary antenatal care from May 2008 to May 2010. The study design has been presented in detail elsewhere (18). The women were included at <20 weeks’ gestation (Mean=15, standard deviation (s.d.)=3) and reexamined at 28 ± 2 weeks’ gestation. All women were given oral and written information about the Stork Groruddalen project when attending the Child Health Clinics for antenatal care and invited to participate. The women who chose to participate gave informed written consent at inclusion, on behalf of themselves and their offspring.

The women were eligible if they were: (1) living in one of the three city districts, (2) would give birth at the study hospitals, (3) in gestational week < 20, (4) not suffering from diseases necessitating intensive hospital follow-up during pregnancy, (5) not already included in the study with a previous pregnancy lasting > 22 weeks, (6) could communicate (orally) in Norwegian or one of the other eight languages, and (7) were able to provide informed written consent. Overall participation rate was 74% and the study cohort was representative for women attending the Child Health Clinics with respect to ethnicity and age, and fairly representative with respect to parity. Questionnaire data (through interview) and fasting blood samples were collected at both visits by specially trained study midwifes, supported by a professional interpreter and translated material when needed. At birth the weight of the baby was measured on calibrated electronic scales and umbilical cord serum was collected and frozen directly on -80°C.

### Maternal Factors

The participants’ ethnic origin was defined by her country of birth. If the participant’s mother was born outside Europe or North America, country of origin was defined by the participant’s mother’s country of birth (18). All women with non-European ancestry were either born or had mothers born outside Europe.

Maternal height was measured at enrollment to the nearest 0.1 cm with a fixed stadiometer (18). Body weight and estimated body fat were measured with a bioelectrical impedance analysis (BIA) scale (Tanita-BC 418 MA, Tanita Corporation, Tokyo, Japan) at both visits with light clothing and without shoes. Pre-pregnant body mass index (BMI, weight (kg)/height (m)^2^) was calculated using pre-pregnant weight (self-reported at inclusion), to the nearest kg, and measured height.

Parity was categorized as “primiparous” or “parous” (at least one previous pregnancy lasting more than 22 weeks). Maternal age was recorded in years at enrolment. Maternal present socioeconomic position was a score derived from a principal components analysis (PCA) of 11 different demographic variables (19). The variables contributing most to the score, were individual level data about education, occupational class and employment status, and household variables as own or renting tenure and rooms per person in the household. This score was normally distributed (mean=0, median=0.1, SD=1 range:-2.91 to 2.59). Maternal childhood socioeconomic position was derived from a separate PCA of three sociodemographic variables (family occupational class (highest of mother and father), rooms per person in household and family ownership of car, all referring to maternal age of 10 years), and was also normally distributed.

### Biochemical Analyses

A standard 75 g oral glucose tolerance test (OGTT) was performed at Visit 2 at 28 ± 2 weeks’ gestation. Women were diagnosed with gestational diabetes (GDM) according to the criteria recommended by the World Health Organization (WHO) between 1999-2013 (fasting plasma glucose >7 mmol/L or 2-hour glucose >7.8nmol/L) and given life style advise or specialist follow-up according to guidelines. However, for the present study we have defined GDM by the WHO 2013 criteria (fasting glucose ≥5.1 mmol/l or 2-h glucose ≥8.5 mmol/l) (20).

Fasting triglycerides, HDL- and total cholesterol (all measured in mmol/L) were analysed in serum, consecutively at each study visit, with a colorimetric method (Vitros 5.1 FS, Ortho clinical diagnostic at the routine laboratory at Akershus University Hospital). Serum 25-OH vitamin D was analysed by competitive RIA (Dia-Sorin) at the Hormone Laboratory, Oslo University Hospital. LDL-cholesterol was calculated using Friedewald`s formula (21) as follows: LDL-cholesterol = total cholesterol – HDL-cholesterol – (0.45 x triglycerides) mmol/L, which correlate well with directly measured LDL both early and late in pregnancy (r=0.97) (22). No women used lipid-lowering agents at any visit. Maternal and umbilical cord S-leptin (pg/ml) was analyzed in 2012 from biobanked (-80°C) material with the Luminex xMAP technology (Millipore Corporation, Billerica, MA, USA) at the Hormone Laboratory, Oslo University Hospital. Serum vitamin B12 and folate were measured from biobanked material in 2016, using electrochemiluminescence (ECLIA) assays, Roche, at Medical Biochemistry, Oslo University Hospital.

### Placental Macroscopic Examinations and Processing

Placentas were refrigerated immediately after birth. The next working day placentas were macroscopically examined by a placental pathologist according to a standardized protocol, including weight before and after removal of membranes and cord, length (largest diameter), width (smallest diameter), thickness (central) and description of pathological changes. Further, section samples from the umbilical cord, membranes and two cross sections from macroscopic normal looking placental tissue in the central part of the disc, cross sections from pathological looking tissue and additional sections from the maternal plate, were taken. Tissue sections were fixed in buffered formalin and routinely processed and paraffin embedded (FFPE). For this study, FFPE tissue from the cross-sections of central normal looking placenta were used.

### Placental Tissue Sample Preparation

DNA and RNA were purified using the Allprep DNA/RNA FFPE Kit (Qiagen, Hilden, Germany) according to manufacturer’s instructions. In the present study only DNA was further analyzed. DNA quantity was obtained using Qubit™ dsDNA HS Assay Kit (Invitrogen,Thermo Fisher Scientific, Waltham, MA, USA). Samples were stored at -20°C until further processed. Bisulfite conversion was performed using the EpiTect Fast DNA Bisulfite Kit (Qiagen) according to manufacturer’s instructions. In total, 500 ng of DNA was bisulfite converted for subsequent pyrosequencing analyses, with exception of samples where purified DNA concentrations were less than 20 ng/µl, where 300 ng of DNA was used.

### Pyrosequencing

DNA methylation analyses of *LEP* and 13 CpG sites were performed as described by Bekkering et al. (23) The CpG annotation is also according to Bekkering et al. and the CpG site locations relative to *LEP* transcription start site (TSS) and nucleotide position are given in **Table 1**. Briefly, the PCR and sequencing primers were ordered using PyroMark Custom Assay (Qiagen). PCR amplification of the bisulfite converted DNA was performed using the PyroMark PCR Kit (Qiagen) and 1µl bisulfite template, and cycling conditions as described in the PyroMark PCR Kit protocol.

**Table 1:** The CpG site location relative to *LEP* transcription start site (TSS) and nucleotide position.

CpG	Location relative to TSS	GRCh38.p7 CpG nucleotide position
CpG1	-127	chr7:128.241.151
CpG2	-123	chr7:128.241.155
CpG3	-118	chr7:128.241.160
CpG4	-115	chr7:128.241.163
CpG5	-100	chr7:128.241.178
CpG6	-95	chr7:128.241.183
CpG7	-85	chr7:128.241.193
CpG8	-74	chr7:128.241.204
CpG9	-71	chr7:128.241.207
CpG10	-62	chr7:128.241.216
CpG11	-51	chr7:128.241.227
CpG12	-38	chr7:128.241.240
CpG13	-33	chr7:128.241.245

Pyrosequencing was performed using the PyroMarkQ48 Autoprep (Qiagen) according to manufacturer’s instructions. All reactions were conducted in duplicates and for each sequencing run three controls, non-template (RNase-free water), unmethylated DNA and methylated DNA, were included. The two latter from the EpiTect PCR Control DNA Set (Qiagen). All duplicates were restricted to a minimum difference in % methylation of < 5%. If the criteria were not met the samples were re-run. The mean percentage DNA methylation was used per sample. The sequencing data for all 13 CpG sites in *LEP* were quality control checked by the PyroMarkQ48 Autoprep software and the percentage of DNA methylation were calculated. For samples where CpG site(s) did not pass quality control, analyses were repeated until satisfactory results were obtained. Two samples were excluded as they repeatedly failed quality control.

### Statistical Methods

Descriptives are given as mean (SD), median (IQR) and n (%) as appropriate. We examined the correlation between the methylation of the different CpGs using scatterplots and the Pearson’s correlation coefficient. Univariate linear regression analyses were first used to explore associations of maternal factors, placental characteristics and circulating leptin with *LEP* methylation (CpG 1-13). Based on these results we further performed multivariate general linear model analyses to explore the independent effects of the maternal factors of most interest according to our aim (ethnicity, GDM, LDL-cholesterol and fat mass) on*LEP* methylation, adjusting for clinically important covariates; age, parity, socioeconomic status and height. In a final model we also adjusted for maternal vitamin B12, folate and 25-OH vitamin D levels. We assessed potential interactions with ethnicity by examining scatterplots and by performing our analyses stratified by ethnic group; first univariate and then in the fully adjusted models. We also entered interaction terms between ethnicity*GDM or ethnicity*LDL-cholesterol, one by one into the fully adjusted models. In sensitivity analyses we replaced “total fat mass at enrollment” as the measure of maternal adiposity with either pre-pregnant BMI, total fat mass at 28 weeks’ gestation or maternal sum of skinfolds, with similar results.

A priori, we planned to draw conclusions based on effect estimates and their CIs, rather than statistical tests using an arbitrary P-value cut-off. Nevertheless, in tables p-values <0.05 are written in bold and in figures the point estimates are marked with * reflecting the precision of the estimate (* when p<0.05 but >0.01, ** when p ≤ 0.01 but >0.001 and *** when p<0.001). All P-values given are uncorrected for multiple testing, i.e. we did not take into account the number of CpG sites tested (*N*=13).

## Results

### Sample Characteristics

Eligible for the present study were ethnic Western European and South Asian mothers participating in the STORK-Groruddalen cohort, with singleton pregnancies, gestational age ≥35 weeks and a valid weight at birth, a macroscopic placenta examination performed, and data regarding GDM from an oral glucose tolerance test (OGTT), offered to all study participants (**Figure S1**, Flow chart). From these we randomly chose 80 placentas, 40 European (36 with Norwegian and 4 with other Western European background) and 40 South Asian (26 with Pakistani and 14 with Sri Lankan or Indian background), with similar numbers of boys and girls in both groups.

Characteristics of the study participants are presented in **Table 2**. South Asian mothers were slightly younger, had a lower early life and present socioeconomic position and were shorter. They also had lower levels of vitamin B12, folate and 25-OH vitamin D in early pregnancy. Mean body fat did not differ significantly, while 2-hour glucose levels from the OGTT at 28 weeks’ gestation was slightly higher and LDL-cholesterol was lower in the South Asians. Differences between the two ethnic groups reflected the differences previously reported in the total cohort (n=823) (6, 19, 20). ^+(Waage,et. al. submitted)^ South Asian mothers also had smaller placentas, represented by a lower weight and smaller length and width, but a similar thickness, and birthweight of the offspring was lower (**Table 2**).

**Table 2:** Maternal and placental characteristics.

	n	Total sample	n	Western European	n	South Asian	p
**Maternal characteristics**							
Gestational week at enrollment	80	15.0 (3.3)	40	14.0 (2.2)	40	**16.0 (3.8)**	**0.007**
Gestational week at OGTT^a^	80	28.2 (1.1)	40	28.2 (1.3)	40	28.2 (0.9)	0.8
Age (years), mean (sd)	80	29.5 (4.6)	40	30.7 (4.5)	40	**28.3 (4.3)**	**0.02**
Parity (number of previous births)	80		40		40		0.2
Nullipara		30 (37%)		16 (40%)		14 (35%)	
Para 1		36 (45%)		20 (50%)		16 (40%)	
Para 2+		14 (18%)		4 (10%)		10 (25%)	
Born in Norway	80	40 (50%)	40	36 (90%)	40	**4 (10%)**	**<0.001**
Childhood socioeconomic score^b^	79	0.02 (1.0)	40	0.72 (0.77)	39	**-0.60 (0.67)**	**<0.001**
Present socioeconomic score^c^	80	0.2 (0.8)	40	0.5 (0.7)	40	**-0.2 (0.8)**	**0.001**
Any smoking at OGTT	80	0	40	0	40	0	–
Height (cm)	80	164.7 (5.8)	40	168.0 (5.4)	40	**161.3 (4.0)**	**<0.001**
Prepregnant BMI (kg/m2)	78	24.1 (3.9)	39	24.7 (3.7)	39	23.5 (4.0)	0.2
Total fat mass (kg) at enrollment^d^	80	23.2 (8.4)	40	24.2 (8.5)	40	22.1 (8.2)	0.3
Sum of skinfolds (mm) at enrollment^e^	73	72.2 (19.6)	36	69.3 (20.5)	37	75.0 (18.5)	0.2
**Nutritional factors (at enrollment)**							
Vitamin B12 (pmol/L)	79	247 (86)	39	268 (93)	40	**227 (76)**	**0.03**
Folate (nmol/L)	79	23.7 (10.9)	39	28.6 (9.6)	40	**18.9 (10.0)**	**<0.001**
25OH-Vitamin D (nmol/L)	79	50.8 (27.7)	39	70.0 (22.7)	40	**32.1 (17.4)**	**<0.001**
**Glucose measures (at OGTT)**							
Gestational diabetes^f^	80	34 (43%)	40	15 (38%)	40	19 (48%)	0.4
Fasting glucose (mmol/L)	80	4.9 (0.6)	40	4.9 (0.6)	40	5.0 (0.5)	0.4
2-hour plasma glucose (mmol/L)	79	6.1 (1.4)	40	5.7 (1.2)	39	6.4 (1.5)	**0.02**
**Lipids (at OGTT)**							
Fasting HDL (mmol/L)	79	1.9 (0.4)	39	2.0 (0.5)	40	1.8 (0.4)	0.2
Fasting LDL (mmol/L)	78	3.5 (0.9)	39	3.9 (0.8)	39	**3.2 (0.9)**	**0.001**
Fasting Triglycerides (mmol/L)	79	2.0 (0.8)	39	2.0 (0.8)	40	2.0 (0.7)	0.8
**Placental characteristics**							
Weight (g) excl. cord and membr.	80	495 (115)	40	520 (116)	40	**469 (110)**	**0.04**
Thickness (cm)	77	2.5 (0.5)	38	2.6 (0.5)	39	2.6 (0.5)	0.9
The larger diameter (cm)	79	18.5 (2.1)	39	19.1 (2.3)	40	**18.0 (1.8)**	**0.02**
The smaller diameter (cm)	79	16.6 (2.0)	39	17.0 (1.8)	40	**16.1 (2.0)**	**0.03**
Morphological changes	80	14 (18%)	40	5 (13%)^g^	40	9 (23%)^h^	–
**Birth characteristics**							
Gestational age (days)	80	282 (10)	40	283 (10)	40	279 (10)	0.1
Birthweight (g)	80	3413 (550)	40	3583 (541)	40	**3242 (511)**	**0.005**
**Serum leptin**							
S-leptin (pg/ml) at enrollment	80	1411 (912, 1998)	40	1116 (802, 1752)	40	1727 (1213, 2137)	0.07
S-leptin (pg/ml) at OGTT	78	1925 (1158, 3055)	40	1708 (970, 2457)	38	2225 (1380, 3089)	0.2
Umbilical cord S-leptin (pg/ml)	72	1980 (1056, 3463)	37	1982 (879, 3078)	35	1945 (1148, 4023)	0.7

Numbers are mean (sd), n (%) or median (IQR) as appropriate. Differences between the two ethnic groups were assesses using Pearson Chi-Square tests or t-tests, as appropriate. P-values for such possible differences are given in the right column. Significant differences are marked in bold.

a OGTT = Oral glucose tolerance test performed at 28 ± 2 weeks’ gestation (glucose measured fasting and 2 hours after drinking 75g glucose; to diagnose gestational diabetes)

GDM classified by WHO 2013 criteria = Fasting glucose ≥ 5.1 mmol/L or 2-hour glucose ≥ 8.5 mmol/L.

b Score extracted form a Principal Components Analyses of 3 demographic variables reflecting maternal socioeconomic status at age 10 years. Mean=0, median=0.1, SD=1 range:-2.91 to 2.59).

c Score extracted form a Principal Components Analyses of 11 demographic variables reflecting maternal socioeconomic status at enrolment.

d Measured with a bioelectrical impedance analysis (BIA) scale (Tanita-BC 418 MA).

e Sum of suprailiac, triceps and subscapular skinfolds, measured by Holtain T/W Caliper 0-48mm (Holtain Ltd., Crymych; UK).

f As defined by the WHO 2013 criteria (fasting glucose ≥5.1 mmol/l or 2-h glucose ≥8.5 mmol/l).

g Two placentas with signs of chorioamnionitis, three placentas with small infarctions (<5% of volume).

h One placenta with signs of chorioamnionitis, six placentas with small infarctions (five < 5% of volume, one 25% of volume), one placenta with signs of vilitis.

### Associations Between Maternal Factors and Placental *LEP* Methylation

**Figure 1:**
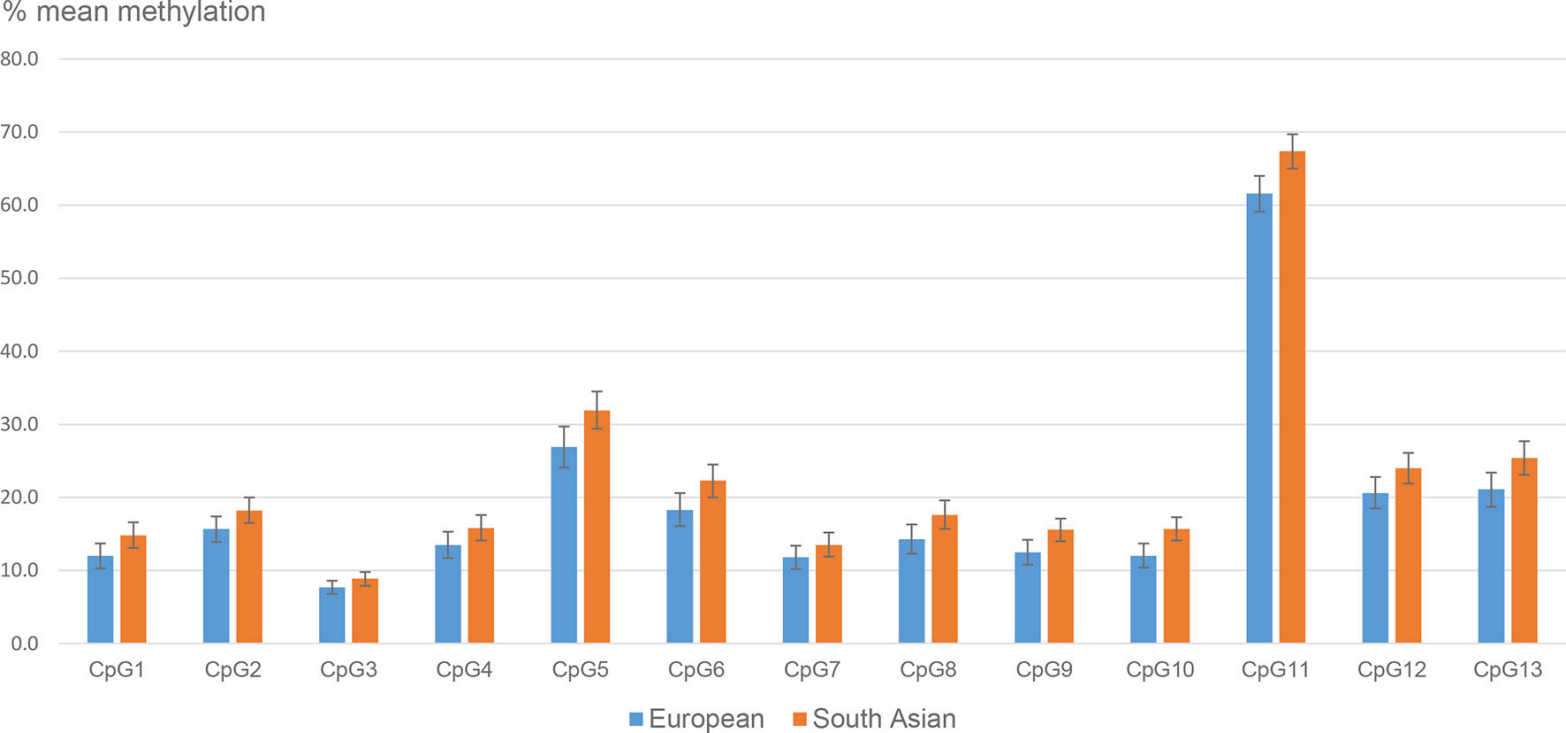
Mean placental *LEP* methylation (95% CI) in ethnic European and South Asian women.

The mean *LEP* methylation level in the placental tissue differed considerably between the 13 *LEP* CpGs, ranging between 8% (CpG3) and 63% (CpG11) (**Table S1**). The methylation of the different CpGs were highly correlated with each other (Pearson’s correlation coefficient >0.8), except for CpG11, which was less correlated with CpG 1, 2, 3, 4, 7, 9 and 10, and for CpG5, which was less correlated with CpG 2 and 3 (correlation coefficients between 0.5-0.7) (data not shown). For all CpGs, the mean placental methylation level was numerically higher in South Asians than in Europeans, and the differences were significant for 10 out of 13 CpGs (**Figure 1** and **Tables S2A**–**E**). The largest difference was observed for CpG11 (mean difference: 5.8% (95% CI: 2.4, 9.2), p<0.001), followed by CpG5 (5.0% (1.3, 8.8), p=0.01).

Measures of placental size (weight, length, width or thickness) were not consistently associated with *LEP* methylation (**Tables S2A**–**E**). We also found no associations with any of the measures of maternal adiposity (total fat mass at enrollment, pre-pregnant BMI, total fat mass at 28 weeks’ gestation or maternal sum of skinfolds), or with circulating s-leptin in maternal nor umbilical cord venous blood (**Tables S2A**–**E**), in any of the two ethnic groups (data not shown).

In univariate analyses GDM was associated with higher placental *LEP* methylation, with higher levels for 8 out of 13 CpGs (**Tables S2A**–**E**). The strongest association was seen for CpG5 (5.2% (1.4, 9.0), p=0.008, **Table S2B**), followed by CpG12 (4.6 (1.6, 7.5), p=0.003, **Table S2D**). However, while univariate nominal associations with GDM were observed for 11 out of 13 CpGs in South Asians, only smaller, non-significant trends were seen in Europeans (**Table 3**). Exploration of scatteplots and univariate analyses did not indicate similar differential effects of LDL-cholesterol in the two ethnic groups.

**Table 3:** Mean % placental *LEP* methylation (95% CI) of CpG 1-13 in ethnic European and South Asian women with and without GDM.

	Europe			South Asia		
	non-GDM	GDM		non-GDM	GDM	
	n = 25	n = 15	p	n = 21	n = 19	p
CpG1	11.5 (9.4, 13.6)	12.8 (10.1, 15.5)	0.5	12.6 (10.4, 14.9)	17.3 (14.9, 19.7)	**0.007**
CpG2	15.5 (13.3, 17.7)	15.9 (13.2, 18.6)	0.8	16.5 (14.2, 18.8)	20.3 (17.8, 22.7)	**0.04**
CpG3	7.2 (6.1, 8.4)	8.4 (6.9, 9.9)	0.2	8.1 (6.9, 9.4)	9.8 (8.4, 11.3)	0.08
CpG4	12.8 (10.5, 15.0)	14.5 (10.5, 15.0)	0.4	14.3 (11.9, 16.6)	17.8 (15.2, 20.4)	**0.04**
CpG5	26.0 (22.7, 29.3)	28.7 (24.1, 33.2)	0.4	29.1 (25.8, 32.4)	35.4 (31.7, 39.1)	**0.01**
CpG6	18.4 (15.5, 21.3)	18.3 (14.9, 21.7)	0.9	20.0 (17.1, 22.9)	25.3 (22.0, 28.6)	**0.02**
CpG7	11.2 (9.1, 13.3)	12.7 (10.2, 15.2)	0.4	12.4 (10.3, 14.6)	14.9 (12.4, 17.3)	0.1
CpG8	14.1 (11.7, 16.6)	14.7 (11.5, 17.8)	0.8	15.7 (13.1, 18.3)	19.9 (17.1, 22.7)	**0.03**
CpG9	12.1 (10.1, 14.1)	13.2 (10.5, 15.8)	0.5	13.7 (11.6, 15.8)	17.7 (15.5, 20.0)	**0.02**
CpG10	11.8 (9.8, 13.8)	12.4 (9.9, 14.9)	0.7	13.7 (11.6, 15.8)	17.8 (15.6, 20.1)	**0.01**
CpG11	60.7 (57.7, 63.8)	62.9 (59.1, 66.7)	0.4	65.3 (62.1, 68.5)	69.7 (57.7, 63.8)	**0.03**
CpG12	19.5 (16.9, 22.0)	22.5 (19.2, 25.7)	0.2	21.5 (18.9, 24.1)	27.3 (24.3, 30.3)	**0.004**
CpG13	20.8 (17.8, 23.9)	21.6 (18.0, 25.2)	0.7	22.9 (20.0, 25.9)	28.4 (25.1, 31.7)	**0.02**

p-values represents the significance level for the difference between GDM and non-GDM from univariate general linear models (similar to t-tests). Significant differences are written in bold.

Regarding maternal lipids, higher fasting LDL-cholesterol levels at 28 weeks’ gestation was associated with lower placental *LEP* methylation in 12 out of 13 CpGs (**Tables S2A**–**E**). The strongest association was seen for CpG11, where one mmol/L increase in LDL-cholesterol was associated with 3.6% (-5.5, -1.4) lower methylation (p<0.001, **Table S2D**), followed by CpG5 (-3.2% (-5.3, -1.2, p=0.003), **Table S2B**). HDL-cholesterol was not consistently associated with *LEP* methylation, although nominally negatively associated with CpG11 methylation (p=0.03). For fasting triglycerides, we observed no significant associations with DNA methylation for any of the 13 CpG sites in *LEP*.

Using multivariate linear models we further examined the independent effects of ethnicity and metabolic factors on placental *LEP* methylation. As the strongest associations with ethnicity were found for CpG11, 5 and 13, results from multivariate are shown for these three CpGs in **Tables 4A–C**. However, similar results are shown for all CpGs in **Table S3**. As described above, South Asian ethnic origin and GDM were positively and LDL-cholesterol negatively associated with the *LEP* methylation level in univariate analyses. After mutual adjustments, and adjustments for maternal age, height, early life socioeconomic position and parity, GDM and LDL-cholesterol remained significantly associated with the methylation level, while the effect estimate for ethnicity was attenuated and no longer significant (**Table 4**, Model 1). When further adjusting for maternal vitamin D, B12 and folate status, the association with ethnicity disappeared (**Table 4**, Model 2), the association with GDM was somewhat attenuated and only borderline significant while the association with LDL-cholesterol remained significant. In fully adjusted models higher maternal folate was associated with higher *LEP* metylation.

**Table 4:** Associations between maternal factors and placental *LEP* CpG5, CpG11 and CpG13 methylation.

**Table 4a | Associations with CpG5 methylation.**						
	Univariate		Model 1		Model 2	
	β (95% CI)	p	β (95% CI)	p	β (95% CI)	p
South Asian ethnicity	5.0 (1.3, 8.8)	**0.01**	2.9 (-3.6, 9.4)	0.4	1.4 (-5.9, 8.7)	0.7
Gestational diabetes	5.2 (1.4, 9.0)	**0.008**	4.8 (0.6, 8.9)	**0.03**	4.0 (-0.1, 8.1)	0.05
Fat mass (kg)	0.0 (-0.2, 0.3)	0.9	-0.1 (-0.4, 0.2)	0.6	-0.0 (-0.3, 0.3)	0.9
LDL cholesterol	-3.2 (-5.3, -1.2)	**0.003**	-2.1 (-4.3, 0.2)	**0.07**	-2.8 (-5.0, -0.6)	**0.02**
Vitamin B12	-0.01 (-0.04, 0.02)	0.5			-0.02 (-0.05, 0.25)	0.2
Folate	-0.00 (-0.19, 0.18)	1			0.26 (0.05, 0.48)	**0.02**
Vitamin D	-0.08 (-0.15, -0.01)	**0.02**			-0.07 (-0.17, 0.04)	0.2
Table 4b | Associations with CpG11 methylation.						
	**Univariate**		**Model 1**		**Model 2**	
	**β (95% CI)**	**p**	**β (95% CI)**	**p**	**β (95% CI)**	**p**
South Asian ethnicity	5.8 (2.4, 9.2)	**0.001**	3.8 (-1.5, 9.1)	0.2	0.7 (-5.1, 6.5)	0.8
Gestational diabetes	3.8 (0.2, 7.3)	**0.04**	3.9 (0.2, 7.5)	**0.04**	2.7 (-0.9, 6.3)	0.1
Fat mass (kg)	-0.1 (-0.3, 0.2)	0.6	-0.1 (-0.4, 0.1)	0.4	-0.1 (-0.3, 0.2)	0.6
LDL cholesterol	-3.6 (-5.5, -1.8)	**<0.001**	-2.5 (-4.4, -0.5)	**0.02**	-3.1 (-5.1, -1.2)	**0.002**
Vitamin B12	-0.01 (-0.04, 0.01)	0.2			-0.02 (-0.04, 0.01)	0.1
Folate	-0.07 (-0.24, 0.10)	0.4			0.15 (-0.02, 0.32)	0.09
Vitamin D	-0.10 (-0.17, -0.04)	**0.001**			-0.07 (-0.16, 0.02)	0.1
Table 4c | Associations with CpG13 metylation.						
	**Univariate**		**Model 1**		**Model 2**	
	**β (95% CI)**	**p**	**β (95% CI)**	**p**	**β (95% CI)**	**p**
South Asian ethnicity	4.2 (0.9, 7.5)	**0.01**	4.3 (-0.9, 9.6)	0.1	3.6 (-2.2, 9.5)	0.2
Gestational diabetes	3.4 (0.05, 6.8)	**0.04**	4.4 (0.8, 8.0)	**0.02**	3.4 (-0.3, 7.1)	0.07
Fat mass (kg)	-0.1 (-0.3, 0.1)	0.3	-0.2 (-0.4, 0.02)	0.07	-0.1 (-0.4, 0.1)	0.4
LDL cholesterol	-2.4 (-4.3, -0.6)	**0.01**	-1.5 (-3.4, 0.5)	**0.1**	-2.2 (-4.2, -0.3)	**0.03**
Vitamin B12	-0.01 (-0.04, 0.01)	0.2			-0.02 (-0.04, 0.01)	0.2
Folate	0.03 (-0.13, 0.19)	0.7			0.22 (0.04, 0.40)	**0.02**
Vitamin D	-0.05 (-0.11, 0.01)	0.1			-0.02 (-0.11, 0.07)	0.7

Betas are effect estimates from univariate and multivariate general linear models, adjusting for covariates. Significant effects (p < 0.05) are written in bold.

Model 1: Variables included in the model: ethnicity, age, height, early life socioeconomic position, parity, gestational diabetes, total fat mass and LDL-cholesterol.

Model 2: Variables included in the model: ethnicity, age, height, early life socioeconomic position, parity, gestational diabetes, total fat mass, LDL-cholesterol, vit B12, folate and 25-OH Vit D.

Last, we checked for interactions with ethnicity, by entering interaction terms ethnicity* GDM or ethnicity*LDL-cholesterol one by one into the fully adjusted models. No significant interactions were observed (p>0.3 for all, and >0.7 for CpG5 and CpG11). We also performed multivariate analyses stratified by ethnic group. Although not significant in all, effect estimates for GDM, LDL-cholesterol, adiposity and nutritional factors were similar across the two ethnic groups (data not shown). In supplementary analyses, exchanging GDM with maternal fasting or 2-hour glucose as explanatory factors, we found similar results as when using GDM (**Tables S4A**–**C**).

## Discussion

As far as we are aware, this is the first study exploring ethnic differences in placental *LEP* methylation, and relationships with both maternal glycaemia, lipid levels and adiposity. We found that South Asian ethnic origin and maternal GDM were associated with higher placental *LEP* methylation, while higher LDL-cholesterol was associated with lower *LEP* methylation. The strongest associations with these three maternal factors were found for CpG11 and CpG5, which are known binding sites for important transcription factors. Ethnic differences were, however, strongly attenuated and no longer significant when adjusting for metabolic factors and vitamin status. In contrast, measures of placental size, maternal adiposity or circulating leptin levels were not associated with placental *LEP* methylation.

Leptin is involved in the regulation of multiple aspects of maternal metabolic homeostasis (7). Furthermore, leptin has been shown to also be important for placentation and maternal–fetal exchange processes (5). We did not find any associations between maternal or fetal circulating levels of leptin and placental *LEP* methylation. Hence, placental *LEP* methylation seems to be related to other factors than maternal and offspring leptin levels.

To the best of our knowledge, no other studies have so far investigated variations in placental *LEP* methylation across different ethnic groups. Limited information also exists from other tissues. A study from US comparing whole blood *LEP* methylation in adults and children with Northern European vs Vietnamese (East Asian) origin showed a lower methylation level in ethnic Vietnamese participants (24). Interestingly, in line with our data they also found the largest difference in the CpG-site that corresponds to our CpG11. However, the relationship to ethnicity was in the opposite direction compared to our findings in placentas from South Asian mothers. We can only speculate as to whether this merely reflects that methylation is tissue-specific, or whether it could be related to differences between these two Asian sub-groups.

Consistently, higher DNA methylation levels in South Asians were observed across all CpG sites investigated in the present study, suggesting a general hypermethylation of *LEP* in placentas of South Asians compared with individuals of European descent. The mechanistic background remains elusive, but could partly be due to genetic factors, as genetic variation may partly influence DNA methylation, e.g. by SNP markers introducing or deleting CpG sites (25, 26). However, people belonging to different ethnic groups may also differ on several other environmental factors not captured by genetic ancestry. For example, changes in the methylation of *LEP* have been observed in blood cells in adults exposed to famine when *in-utero* (27). In our study, 90% of the women with South Asian ancestry were born in Pakistan, India or Sri Lanka, classified as low or middle income countries, and had a lower socioeconomic position in childhood, compared with women of European origin. Although we did not find significant associations with maternal height or early life socioeconomic position, this could potentially have induced adaptive effects on regulatory mechanisms within the placenta.

In our cohort of pregnant women with expected normal pregnancies, all women were offered screening for GDM, and the majority hence had a relatively mild GDM (20). Nevertheless, we found that GDM was associated with higher *LEP* methylation. This is in line with some previous studies (17, 28). One study by Lesseur et al., obtaining GDM status from medical charts, found that placentas from mothers with GDM had 2.5% higher *LEP* methylation levels than in mothers not diagnosed with GDM (28). This effect estimate was slightly smaller than in our study. In another study by Bouchard et al. they found that in women with impaired glucose tolerance, placental leptin gene DNA methylation levels were associated with maternal 2-h glucose levels. However, this association depended on the placental site the tissue was sampled from (10). The different methodology makes direct comparison with our findings difficult and illustrates the complexity involved in these relationships.

In contrast to our findings, Lesseur et al. reported an association between maternal obesity and placental *LEP* methylation, and that this was largely mediated through an effect on GDM (28). Further, a study from France observed placental hypermethylation and hypoexpression of *LEP* in relation with maternal obesity (8), while a study from Spain did not find any obesity-related changes in placental *LEP* expression (29). We found no differences in placental *LEP* methylation across various levels of maternal adiposity. These findings were similar in the two ethnic groups and across different measures of adiposity, suggesting that adiposity is not the primary explanation for the observed associations with ethnicity and glucose- and lipid metabolism.

We have not been able to find any previous studies exploring associations between maternal lipid/lipoprotein levels and placental *LEP* methylation. A study by Houde et al. did show that in severely obese non-pregnant adults, LDL-cholesterol levels were positively associated with *LEP* methylation levels both in whole blood and in adipose tissue, suggesting that LDL-cholesterol might be involved in the epigenetic regulation of leptin (30). However, the positive associations were, in contrast to the negative association observed in placental tissue in our study. LDL-cholesterol is possibly the most important source of cholesterol for the fetus (3). A recent Norwegian *in vivo* study showed that fetal cholesterol uptake was related to the uteroplacental uptake of cholesterol from LDL-cholesterol, suggesting that the placenta influences maternal-fetal cholesterol transfer (31). Some previous studies have indicated that leptin plays a role in the regulation of placental glucose and amino-acid transport, by up-regulating specific placental nutrient transporter isoforms (5). From our finding we could thus speculate that placental leptin may also be involved in the regulation of LDL-cholesterol transport in the placenta.

Interestingly, in the study by Houde et al., as in our study, the strongest associations with LDL cholesterol were found for the two CpG sites that corresponds to our CpG11 and CpG5. In our study the strongest relations with ethnicity and GDM were also seen for CpG11 and CpG5. Methylation of these two CpGs, and in particular CpG11, were less correlated with the metylation of other CpGs. These two CpGs are located at binding sites for known transcription factors (32). CpG5 is located at the binding site for the specificity protein 1 (SP1), known to be involved in the *LEP* gene expression regulation in adipocytes (32, 33), especially in the induction of leptin by insulin-stimulated glucose metabolism as reported by Moreno-Aliaga et al. (34) Further, CpG11 is located at a binding site for CCAAT/enhancer binding protein-α (CEBPA). The gene coding for CEBPA is considered a core regulatory gene in the control of adipogenesis (11). Binding of CEBPA at this site highly activates the transcription of *LEP*, and methylation of this site has been shown to repress transcription of the *LEP* gene (32, 35). Our results support that also in placental tissue it is likely that these sites are subject to regulatory mechanisms and changes in gene expression affected by maternal glucose metabolism, and thus could have important functions during pregnancy beyond its accepted action on maturation of adipocytes for energy storage (7). However, our findings further suggest that the regulation of *LEP* expression in placental tissue differs from blood and adipose tissue.

In univariate stratified analyses we found that effect estimates for the association between GDM and placental *LEP* methylation were nominally larger, and only significant for South Asians. We could speculate that this finding reflect that GDM in South Asians to a larger extent represent pre-gestational dysglycemia and hence more “severe” GDM. We had limited statistical power to do formal interaction analyses and to test direct vs. indirect effects of the different exposures. However, interaction analyses and stratified analyses in fully adjusted models did not suggest that the effects of GDM, LDL-cholesterol and nutritional factors differed between the two ethnic groups. This indicate that the suggested stronger effect of GDM in South Asians from univariate analyses are mainly related to differences in other covariates.

In our study, South Asian mothers had lower levels of vitamin D, B12 and folate than ethnic European mothers in early pregnancy. Folate and vitamin B12 are key factors in generating methionine that represents a major source of S-adenosylmethionine (SAM), the major methyl-donating substrate for DNA methylation, and hence strongly influences DNA methylation levels (36). Also, vitamin D has effects on genome wide DNA methylation levels and was recently reported to influence long-chain polyunsaturated fatty acids and oxidative stress in the placenta *via* one-carbon metabolism (37). The relations between nutritional factors and the methylation of regulatory cytokines in placenta is likely very complicated, where not only the level of each nutritional factor itself, but also the combination of different factors may be important (38, 39). Differences in vitamin levels could also be proxy measures of other complex life-style factors. Nevertheless, our findings suggest that nutritional factors may be involved in the regulation of key placental cytokines also in generally well-nourished multi-ethnic European populations.

The present study has several strengths. First, we have studied relationships with *LEP* methylation in two distinct ethnic groups, known to have different metabolic phenotypes, participating in the same cohort study, ruling out methodological issues to explain observed ethnic differences. Second, this cohort of pregnant women were followed from early pregnancy, and we have an extensive maternal dataset, including information about early life socioeconomic conditions, lipids/lipoproteins, glucose data from universal screening of GDM, different measures of adiposity and micronutrient status, specifically vitamins known to be involved in the one-carbon metabolism cycle, and more. Nevertheless, there are also important limitations to the study. We had a relatively small sample size, which limited our power; e.g. for interaction analyses and to test direct vs. indirect effects. Although transversal tissue sections from the central placenta will mainly consist of tissue from the fetal compartment (chorionic plate, villi), it could possibly also contain smaller amounts of tissue of maternal origin (intervillous space and basal plate). Moreover, even within fetal tissue different cell types may be present. As methylation is cell-type specific, this could have implications for our results. However, in our study such differences between placental samples should occur completely at random, and would thus not explain the observed associations with ethnicity or lipid and glucose levels. As we observed similar patterns of associations with ethnicity, GDM and LDL-cholesterol across all CpGs, and we consider this study as exploratory and hypothesis generating, we did not correct the p-values for multiple testing, i.e. we did not take into account the number of CpG sites tested (N=13). If we had applied Bonferroni correction for association analyses, the study-specific significance threshold for the analyses would have been lowered to P=0.0038 (0.05/13). Some of the observed associations would hence still be significant.

## Conclusions

The relationships between maternal factors, placental function and fetal development are probably mediated through a complex system likely affected by genomic differences and adaptive processes. Leptin is considered a key regulatory cytokine within the placenta. Our findings suggest that maternal glucose and cholesterol metabolism in pregnancy can alter placental *LEP* metylation, in particular at some CpG sites which are known as binding sites for important transcription factors. Further, our results indicate that differences in maternal metabolism and nutritional status between ethnic Europeans and South Asians could explain most of the ethnic variation in placental *LEP* methylation. Understanding the impact of maternal metabolic and nutritional factors on placental epigenetic marks is particularly important given the current rise in prevalence of obesity, GDM and type 2 diabetes, not least in the South Asian population, and the possible consequences on later-life disease susceptibility.

## Data Availability Statement

The datasets presented in this article are not readily available because of restrictions e.g. their containing information that could compromise the privacy of the study participants. Requests to access the datasets should be directed to the corresponding author.

## Ethics Statement

The studies involving human participants were reviewed and approved by the Regional Committee for Medical and Health Research Ethics for South Eastern Norway. The patients/participants provided their written informed consent to participate in this study.

## Author Contributions

LS, AJ, and KB contributed in the data acquisition. LS and YB planned the study. AM and NL performed the epigenetic laboratory analyses. LS performed the statistical analyses and wrote the manuscript. LS, AM, YB, CY, and CS contributed on the interpretation of the data. All authors critically revised the paper and approved the final manuscript.

## Funding

This work is funded by The South-Eastern Norway Regional Health Authority through a research project grant to LS (Grant number 2017-063). The funder had no role in data collection, analyses or interpretation of data or in writing the paper.

## Conflict of Interest

The authors declare that the research was conducted in the absence of any commercial or financial relationships that could be construed as a potential conflict of interest.

## Publisher’s Note

All claims expressed in this article are solely those of the authors and do not necessarily represent those of their affiliated organizations, or those of the publisher, the editors and the reviewers. Any product that may be evaluated in this article, or claim that may be made by its manufacturer, is not guaranteed or endorsed by the publisher.
