# Supplementary material for: Maternal Glucose and LDL-Cholesterol Levels Are Related to Placental Leptin Gene Methylation, and, Together With Nutritional Factors, Largely Explain a Higher Methylation Level Among Ethnic South Asians
Source: Front Endocrinol (Lausanne). 2021 Dec 24;12:809916. doi: 10.3389/fendo.2021.809916 (PMC8739998; doi:10.3389/fendo.2021.809916)
Supplement: Supplementary file 1 [file DataSheet_1.zip › Table S1.DOCX]

**Table S1.** Mean (sd) % methylation of 13 CpGs of the *LEP* gene

|  | European | |  | South Asian | |
| --- | --- | --- | --- | --- | --- |
|  | Valid cases | Mean (sd) |  | Valid cases | Mean (sd) |
| CpG1 | 37 | 12.0 (5.2) |  | 38 | 14.8 (5.5) |
| CpG2 | 38 | 15.7 (5.2) |  | 39 | 18.2 (5.6) |
| CpG3 | 39 | 7.7 (2.9) |  | 37 | 8.9 (3.0) |
| CpG4 | 38 | 13.5 (5.7) |  | 38 | 15.8 (5.3) |
| CpG5 | 32 | 26.9 (8.1) |  | 38 | 31.9 (31.9) |
| CpG6 | 36 | 18.3 (6.9) |  | 37 | 22.3 (6.8) |
| CpG7 | 36 | 11.8 (5.3) |  | 35 | 13.5 (4.3) |
| CpG8 | 37 | 14.3 (6.1) |  | 39 | 17.6 (6.1) |
| CpG9 | 35 | 12.5 (4.6) |  | 39 | 15.6 (5.2) |
| CpG10 | 38 | 12.0 (4.8) |  | 38 | 15.7 (5.1) |
| CpG11 | 38 | 61.6 (8.2) |  | 39 | 67.4 (6.6) |
| CpG12 | 36 | 20.6 (6.7) |  | 37 | 24.0 (6.3) |
| CpG13 | 34 | 21.1 (6.8) |  | 38 | 25.4 (7.2) |
