# Supplementary material for: Maternal Glucose and LDL-Cholesterol Levels Are Related to Placental Leptin Gene Methylation, and, Together With Nutritional Factors, Largely Explain a Higher Methylation Level Among Ethnic South Asians
Source: Front Endocrinol (Lausanne). 2021 Dec 24;12:809916. doi: 10.3389/fendo.2021.809916 (PMC8739998; doi:10.3389/fendo.2021.809916)
Supplement: Supplementary file 1 [file DataSheet_1.zip › Table S3.DOCX]

**Table S3.** Associations between maternal factors and placental *LEP* methylation.

Table S3a. Associations with CpG1 methylation

|  | Univariate |  |  | Model 1 |  |  | Model 2 |  |
| --- | --- | --- | --- | --- | --- | --- | --- | --- |
|  | β (95% CI) | p |  | β (95% CI) | p |  | β (95% CI) | p |
| South Asian ethnicity | 2.8 (0.4, 5.3) | **0.02** |  | 2.7 (-1.2, 6.6) | 0.2 |  | 2.8 (-1.5, 7.1) | 0.2 |
| Gestational diabetes | 3.3 (0.8, 5.7) | **0.009** |  | 3.6 (0.9, 6.3) | **0.01** |  | 3.1 (0.5, 5.8) | **0.02** |
| Fat mass (kg) | -0.1 (-0.2, 0.1) | 0.6 |  | -0.2 (-0.3, 0.0) | 0.07 |  | -0.1 (-0.3, 0.1) | 0.3 |
| LDL cholesterol | -1.9 (-3.2, -0.5) | **0.007** |  | -1.2 (-2.6, 0.3) | 0.1 |  | -1.6 (-3.1, -0.2) | **0.03** |
| Vitamin B12 | -0.00 (-0.02, 0.01) | 0.7 |  |  |  |  | -0.00 (-0.02, 0.01) | 0.7 |
| Folate | 0.06 (-0.05, 0.18) | 0.3 |  |  |  |  | 0.18 (0.05, 0.31) | **0.007** |
| Vitamin D | -0.04 (-0.08, 0.01) | **0.1** |  |  |  |  | -0.02 (-0.08, 0.05) | 0.6 |

Table S3b. Associations with CpG2 methylation

|  | Univariate |  |  | Model 1 |  |  | Model 2 |  |
| --- | --- | --- | --- | --- | --- | --- | --- | --- |
|  | β (95% CI) | p |  | β (95% CI) | p |  | β (95% CI) | p |
| South Asian ethnicity | 2.6 (0.4, 5.0) | **0.04** |  | 2.0 (-1.9, 6.0) | 0.3 |  | 2.0 (-2.4, 6.3) | 0.4 |
| Gestational diabetes | 2.3 (-0.2, 4.8) | 0.07 |  | 3.0 (0.2, 5.7) | **0.03** |  | 2.5 (-0.2, 5.2) | 0.07 |
| Fat mass (kg) | -0.1 (-0.2, 0.1) | 0.4 |  | -0.1 (-0.3, 0.0) | 0.1 |  | -0.1 (-0.3, 0.1) | 0.4 |
| LDL cholesterol | -1.7 (-3.1, -0.4) | **0.01** |  | -1.2 (-2.7, 0.3) |  |  | -1.8 (-3.2, -0.3) | **0.02** |
| Vitamin B12 | -0.01 (-0.03, 0.01) | 0.3 |  |  |  |  | -0.01 (-0.03, 0.01) | 0.2 |
| Folate | 0.08 (-0.04, 0.19) | 0.2 |  |  |  |  | 0.19 (0.06, 0.32) | **0.006** |
| Vitamin D | -0.03 (-0.08, 0.02) | 0.2 |  |  |  |  | -0.01 (-0.07, 0.06) | 0.8 |

Table S3c. Associations with CpG3 methylation

|  | Univariate |  |  | Model 1 |  |  | Model 2 |  |
| --- | --- | --- | --- | --- | --- | --- | --- | --- |
|  | β (95% CI) | p |  | β (95% CI) | p |  | β (95% CI) | p |
| South Asian ethnicity | 1.2 (-0.2, 2.5) | 0.09 |  | 0.3 (-1.8, 2.5) | 0.8 |  | 0.2 (-2.3, 2.8) | 0.9 |
| Gestational diabetes | 1.5 (0.2, 2.9) | **0.03** |  | 2.0 (0.6, 3.5) | **0.008** |  | 1.8 (0.3, 3.3) | **0.02** |
| Fat mass (kg) | -0.1 (-0.1, 0.0) | 0.2 |  | -0.1 (-0.2, -0.0) | 0.04 |  | -0.1 (-0.2, 0.0) | 0.06 |
| LDL cholesterol | -0.9 (-1.6, -0.1) | **0.02** |  | -0.6 (-1.4, 0.2) | **0.1** |  | -0.8 (-1.7, -0.0) | **0.04** |
| Vitamin B12 | -0.01 (-0.02, 0.00) | 0.07 |  |  |  |  | -0.01 (-0.02, -0.00) | 0.04 |
| Folate | 0.02 (-0.05, 0.08) | 0.7 |  |  |  |  | 0.06 (-0.02, 0.14) | 0.1 |
| Vitamin D | -0.02 (-0.04, 0.01) | 0.2 |  |  |  |  | 0.00 (-0.04, 0.04) | 1 |

Table S3d. Associations with CpG4 methylation

|  | Univariate |  |  | Model 1 |  |  | Model 2 |  |
| --- | --- | --- | --- | --- | --- | --- | --- | --- |
|  | β (95% CI) | p |  | β (95% CI) | p |  | β (95% CI) | p |
| South Asian ethnicity | 2.4 (-0.2, 4.9) | 0.07 |  | 0.4 (-3.8, 4.6) | 0.8 |  | -0.4 (-5.3, 5.0) | 0.9 |
| Gestational diabetes | 2.8 (0.2, 5.3) | **0.03** |  | 3.2 (0.4, 6.0) | **0.03** |  | 2.7 (-0.2, 5.5) | 0.07 |
| Fat mass (kg) | -0.1 (-0.2, 0.1) | 0.3 |  | -0.1 (-0.3, 0.0) | 0.1 |  | -0.1 (-0.3, 0.1) | 0.3 |
| LDL cholesterol | -1.7 (-3.1, -0.3) | **0.02** |  | -1.3 (-2.9, 0.3) | 0.1 |  | -1.7 (-3.3, -0.1) | **0.04** |
| Vitamin B12 | -0.01 (-0.3, 0.01) | 0.3 |  |  |  |  | -0.01 (-0.03, 0.01) | 0.3 |
| Folate | 0.03 (-0.09, 0.15) | 0.6 |  |  |  |  | 0.14 (-0.01, 0.29) | 0.06 |
| Vitamin D | -0.04 (-0.09, 0.00) | 0.06 |  |  |  |  | -0.03 (-0.10, 0.04) | 0.3 |

Table S3e. Associations with CpG5 methylation

|  | Univariate |  |  | Model 1 |  |  | Model 2 |  |
| --- | --- | --- | --- | --- | --- | --- | --- | --- |
|  | β (95% CI) | p |  | β (95% CI) | p |  | β (95% CI) | p |
| South Asian ethnicity | 5.0 (1.3, 8.8) | **0.01** |  | 2.9 (-3.6, 9.4) | 0.4 |  | 1.4 (-5.9, 8.7) | 0.7 |
| Gestational diabetes | 5.2 (1.4, 9.0) | **0.008** |  | 4.8 (0.6, 8.9) | **0.03** |  | 4.0 (-0.1, 8.1) | 0.05 |
| Fat mass (kg) | 0.0 (-0.2, 0.3) | 0.9 |  | -0.1 (-0.4, 0.2) | 0.6 |  | -0.0 (-0.3, 0.3) | 0.9 |
| LDL cholesterol | -3.2 (-5.3, -1.2) | **0.003** |  | -2.1 (-4.3, 0.2) | **0.07** |  | -2.8 (-5.0, -0.6) | **0.02** |
| Vitamin B12 | -0.01 (-0.04, 0.02) | 0.5 |  |  |  |  | -0.02 (-0.05, 0.25) | 0.2 |
| Folate | -0.00 (-0.19, 0.18) | 1 |  |  |  |  | 0.26 (0.05, 0.48) | **0.02** |
| Vitamin D | -0.08 (-0.15, -0.01) | **0.02** |  |  |  |  | -0.07 (-0.17, 0.04) | 0.2 |

Table S3f. Associations with CpG6 methylation

|  | Univariate |  |  | Model 1 |  |  | Model 2 |  |
| --- | --- | --- | --- | --- | --- | --- | --- | --- |
|  | β (95% CI) | p |  | β (95% CI) | p |  | β (95% CI) | p |
| South Asian ethnicity | 3.9 (0.8, 7.1) | **0.02** |  | 2.5 (-2.7, 7.7) | 0.3 |  | 2.2 (-3.4, 7.8) | 0.4 |
| Gestational diabetes | 2.7 (-0.6, 6.0) | **0.1** |  | 3.6 (0.2, 7.1) | **0.04** |  | 3.1 (-0.3, 6.5) | 0.07 |
| Fat mass (kg) | -0.1 (-0.3, 0.1) | 0.1 |  | -0.2 (-0.4, 0.00) | 0.05 |  | -0.1 (-0.3, 0.1) | 0.5 |
| LDL cholesterol | -2.2 (-4.1, -0.4) | **0.02** |  | -1.6 (-3.6, 0.4) | 0.1 |  | -2.3 (-4.2, -0.4) | **0.02** |
| Vitamin B12 | -0.01 (-0.03, 0.01) | 0.4 |  |  |  |  | -0.01 (-0.03, 0.14) | 0.3 |
| Folate | 0.09 (-0.07, 0.24) | 0.3 |  |  |  |  | 0.22 (0.05, 0.39) | **0.01** |
| Vitamin D | -0.05 (-0.11, 0.01) | **0.1** |  |  |  |  | -0.02 (-0.10, 0.07) | 0.7 |

Table S3g. Associations with CpG7 methylation

|  | Univariate |  |  | Model 1 |  |  | Model 2 |  |
| --- | --- | --- | --- | --- | --- | --- | --- | --- |
|  | β (95% CI) | p |  | β (95% CI) | p |  | β (95% CI) | p |
| South Asian ethnicity | 1.7 (-0.6, 3.9) | 0.2 |  | 1.5 (-2.3, 5.2) | 0.4 |  | 0.7 (-3.4, 4.8) | 0.8 |
| Gestational diabetes | 2.0 (-0.3, 4.3) | 0.09 |  | 2.5 (0.02, 5.1) | **0.05** |  | 2.0 (-0.4, 4.5) | 0.1 |
| Fat mass (kg) | -0.08 (-2.1, 0.06) | 0.3 |  | -0.2 (-0.3, 0.01) | 0.07 |  | -0.1 (-0.2, 0.1) | 0.4 |
| LDL cholesterol | -1.5 (-2.8, -0.1) | **0.04** |  | -1.0 (-2.5, 0.5) | **0.2** |  | -1.6 (-3.1, -0.2) | **0.03** |
| Vitamin B12 | -0.01 (-0.03, 0.01) | 0.2 |  |  |  |  | -0.02 (-0.03, 0.00) | 0.09 |
| Folate | 0.06 (-0.04, 0.17) | 0.2 |  |  |  |  | 0.16 (0.04, 0.28) | **0.01** |
| Vitamin D | -0.03 (-0.07, 0.02) | 0.2 |  |  |  |  | -0.03 (-0.09, 0.04) | 0.4 |

Table S3h. Associations with CpG8 methylation

|  | Univariate |  |  | Model 1 |  |  | Model 2 |  |
| --- | --- | --- | --- | --- | --- | --- | --- | --- |
|  | β (95% CI) | p |  | β (95% CI) | p |  | β (95% CI) | p |
| South Asian ethnicity | 3.3 (0.5, 6.1) | **0.02** |  | 3.0 (-1.5, 7.5) | 0.2 |  | 2.4 (-2.6, 7.3) | 0.3 |
| Gestational diabetes | 2.7 (-0.13, 5.6) | **0.06** |  | 3.1 (-0.0, 6.3) | **0.05** |  | 2.3 (-0.9, 5.4) | 0.2 |
| Fat mass (kg) | -0.1 (-0.3, 0.1) | 0.4 |  | -0.2 (-0.4, 0.0) | 0.09 |  | -0.1 (-0.3, 0.1) | 0.3 |
| LDL cholesterol | -1.9 (-3.5, -0.4) | **0.02** |  | -1.2 (-2.9, 0.5) | **0.2** |  | -1.9 (-3.6, -0.3) | **0.03** |
| Vitamin B12 | -0.01 (-0.03, 0.01) | 0.3 |  |  |  |  | -0.02 (-0.04, 0.01) | 0.2 |
| Folate | 0.07 (-0.07, 0.20) | 0.3 |  |  |  |  | 0.23 (0.07, 0.38) | **0.005** |
| Vitamin D | -0.05 (-0.10, 0.01) | **0.07** |  |  |  |  | -0.03 (-0.10, 0.05) | 0.5 |

Table S3i. Associations with CpG9 methylation

|  | Univariate |  |  | Model 1 |  |  | Model 2 |  |
| --- | --- | --- | --- | --- | --- | --- | --- | --- |
|  | β (95% CI) | p |  | β (95% CI) | p |  | β (95% CI) | p |
| South Asian ethnicity | 3.1 (0.8, 5.4) | **0.01** |  | 2.5 (-1.2, 6.2) | 0.2 |  | 0.9 (-3.2, 4.9) | 0.7 |
| Gestational diabetes | 2.9 (0.6, 5.3) | **0.02** |  | 3.1 (0.5, 5.6) | **0.02** |  | 2.7 (0.3, 5.0) | **0.03** |
| Fat mass (kg) | -0.0 (-0.2, 0.1) | 0.6 |  | -0.1 (-0.3, 0.1) | 0.2 |  | -0.0 (-0.2, 0.1) | 0.6 |
| LDL cholesterol | -1.9 (-3.1, -0.6) | **0.005** |  | -1.2 (-2.5, 0.2) | **0.09** |  | -1.8 (-3.1, -0.5) | **0.009** |
| Vitamin B12 | -0.01 (-0.03, 0.00) | 0.1 |  |  |  |  | -0.02 (-0.04, -0.00) | **0.02** |
| Folate | 0.04 (-0.7, 0.15) | 0.5 |  |  |  |  | 0.19 (0.07, 0.32) | **0.003** |
| Vitamin D | -0.04 (-0.09, 0.00) | **0.06** |  |  |  |  | -0.02 (-0.08, 0.04) | 0.5 |

Table S3j. Associations with CpG10 methylation

|  | Univariate |  |  | Model 1 |  |  | Model 2 |  |
| --- | --- | --- | --- | --- | --- | --- | --- | --- |
|  | β (95% CI) | p |  | β (95% CI) | p |  | β (95% CI) | p |
| South Asian ethnicity | 3.6 (1.3, 5.9) | **0.002** |  | 3.2 (-0.4, 6.7) | 0.08 |  | 2.1 (-1.7, 6.0) | 0.3 |
| Gestational diabetes | 2.7 (0.3, 5.0) | **0.03** |  | 3.1 (0.6, 5.6) | **0.01** |  | 2.3 (-0.1, 4.7) | 0.06 |
| Fat mass (kg) | -0.09 (-0.23, 0.05) | 0.2 |  | -0.2 (-0.3, -0.0) | **0.03** |  | -0.1 (-0.3, 0.0) | 0.1 |
| LDL cholesterol | -2.0 (-3.2, -0.7) | **0.003** |  | -1.2 (-2.5, 0.2) | **0.08** |  | -1.7 (-3.0, -0.4) | **0.01** |
| Vitamin B12 | -0.01 (-0.03, 0.01) | 0.2 |  |  |  |  | -0.01 (-0.03, 0.00) | 0.1 |
| Folate | 0.03 (-0.8, 0.14) | 0.6 |  |  |  |  | 0.17 (0.06, 0.29) | **0.004** |
| Vitamin D | -0.6 (-0.10, -0.02) | **0.009** |  |  |  |  | -0.04 (-0.10, 0.03) | 0.2 |

Table S3k. Associations with CpG11 methylation

|  | Univariate |  |  | Model 1 |  |  | Model 2 |  |
| --- | --- | --- | --- | --- | --- | --- | --- | --- |
|  | β (95% CI) | p |  | β (95% CI) | p |  | β (95% CI) | p |
| South Asian ethnicity | 5.8 (2.4, 9.2) | **0.001** |  | 3.8 (-1.5, 9.1) | 0.2 |  | 0.7 (-5.1, 6.5) | 0.8 |
| Gestational diabetes | 3.8 (0.2, 7.3) | **0.04** |  | 3.9 (0.2, 7.5) | **0.04** |  | 2.7 (-0.9, 6.3) | 0.1 |
| Fat mass (kg) | -0.1 (-0.3, 0.2) | 0.6 |  | -0.1 (-0.4, 0.1) | 0.4 |  | -0.1 (-0.3, 0.2) | 0.6 |
| LDL cholesterol | -3.6 (-5.5, -1.8) | **<0.001** |  | -2.5 (-4.4, -0.5) | **0.02** |  | -3.1 (-5.1, -1.2) | **0.002** |
| Vitamin B12 | -0.01 (-0.04, 0.01) | 0.2 |  |  |  |  | -0.02 (-0.04, 0.01) | 0.1 |
| Folate | -0.07 (-0.24, 0.10) | 0.4 |  |  |  |  | 0.15 (-0.02, 0.32) | 0.09 |
| Vitamin D | -0.10 (-0.17, -0.04) | **0.001** |  |  |  |  | -0.07 (-0.16, 0.02) | 0.1 |

Table S3l. Associations with CpG12 metylation

|  | Univariate |  |  | Model 1 |  |  | Model 2 |  |
| --- | --- | --- | --- | --- | --- | --- | --- | --- |
|  | β (95% CI) | p |  | β (95% CI) | p |  | β (95% CI) | p |
| South Asian ethnicity | 3.4 (0.4, 6.3) | **0.03** |  | 3.6 (-0.8, 8.1) | 0.1 |  | 3.1 (-1.9, 8.1) | 0.2 |
| Gestational diabetes | 4.6 (1.6, 7.5) | **0.003** |  | 5.4 (2.4, 8.4) | **0.001** |  | 5.2 (2.1, 8.3) | **0.001** |
| Fat mass (kg) | -0.1 (-0.2, 0.1) | 0.5 |  | -0.2 (-0.4, 0.0) | 0.08 |  | -0.1 (-0.3, 0.13) | 0.4 |
| LDL cholesterol | -2.2 (-3.9, -0.6) | **0.009** |  | -1.2 (-2.9, 0.5) | 0.2 |  | -1.8 (-3.5, -0.0) | **0.05** |
| Vitamin B12 | -0.01 (-0.03, 0.01) | 0.2 |  |  |  |  | -0.01 (-0.03, 0.01) | 0.3 |
| Folate | -0.01 (-0.15, 0.14) | 0.9 |  |  |  |  | 0.13 (-0.02, 0.28) | 0.08 |
| Vitamin D | -0.04 (-0.10, 0.01) | 0.1 |  |  |  |  | -0.00 (-0.08, 0.07) | 0.9 |

Table S3m. Associations with CpG13 metylation

|  | Univariate |  |  | Model 1 |  |  | Model 2 |  |
| --- | --- | --- | --- | --- | --- | --- | --- | --- |
|  | β (95% CI) | p |  | β (95% CI) | p |  | β (95% CI) | p |
| South Asian ethnicity | 4.2 (0.9, 7.5) | **0.01** |  | 4.3 (-0.9, 9.6) | 0.1 |  | 3.6 (-2.2, 9.5) | 0.2 |
| Gestational diabetes | 3.4 (0.05, 6.8) | **0.04** |  | 4.4 (0.8, 8.0) | **0.02** |  | 3.4 (-0.3, 7.1) | 0.07 |
| Fat mass (kg) | -0.1 (-0.3, 0.1) | 0.3 |  | -0.2 (-0.4, 0.02) | 0.07 |  | -0.1 (-0.4, 0.1) | 0.4 |
| LDL cholesterol | -2.4 (-4.3, -0.6) | **0.01** |  | -1.5 (-3.4, 0.5) | **0.1** |  | -2.2 (-4.2, -0.3) | **0.03** |
| Vitamin B12 | -0.01 (-0.04, 0.01) | 0.2 |  |  |  |  | -0.02 (-0.04, 0.01) | 0.2 |
| Folate | 0.03 (-0.13, 0.19) | 0.7 |  |  |  |  | 0.22 (0.04, 0.40) | **0.02** |
| Vitamin D | -0.05 (-0.11, 0.01) | 0.1 |  |  |  |  | -0.02 (-0.11, 0.07) | 0.7 |

The associations between maternal ethnic origin, gestational diabetes (GDM) and LDL-cholesterol -- and placental *LEP* methylation, using multivariate general linear models, adjusting for covariates.

Model 1: Variables included in the model: ethnicity, age, height, early life socioeconomic position, parity, GDM, total fat mass and LDL-cholesterol

Model 2: Variables included in the model: ethnicity, age, height, early life socioeconomic position, parity, GDM, total fat mass, LDL-cholesterol, vit B12, folate and 25-OH Vit D
