# Supplementary material for: Maternal Glucose and LDL-Cholesterol Levels Are Related to Placental Leptin Gene Methylation, and, Together With Nutritional Factors, Largely Explain a Higher Methylation Level Among Ethnic South Asians
Source: Front Endocrinol (Lausanne). 2021 Dec 24;12:809916. doi: 10.3389/fendo.2021.809916 (PMC8739998; doi:10.3389/fendo.2021.809916)
Supplement: Supplementary file 1 [file DataSheet_1.zip › Table S4.DOCX]

**Table S4.** The associations between three different maternal glucose parameters, LDL-cholesterol and ethnic origin and placental *LEP* CpG11 methylation using multivariate general linear models, adjusting for covariates.

**Table S4a.** The associations with maternal ethnic origin, LDL cholesterol and gestational diabetes.

|  | Univariate |  |  | Adjusted^1^ |  |
| --- | --- | --- | --- | --- | --- |
|  | β (95% CI) | p |  | β (95% CI) | p |
| GDM | 3.8 (0.2, 7.3) | **0.04** |  | 2.7 (-0.9, 6.3) | 0.1 |
| LDL cholesterol (mmol/L) | -3.6 (-5.5, -1.8) | **<0.001** |  | -3.1 (-5.1, -1.2) | **0.002** |
| South Asian ethnicity | 5.8 (2.4, 9.2) | **0.001** |  | 0.7 (-5.1, 6.5) | 0.7 |

**Table 4b.** The associations with maternal ethnic origin, LDL-cholesterol and fasting plasma glucose (mmol/L) at 28 weeks' gestation.

|  | Univariate |  |  | Adjusted^1^ |  |
| --- | --- | --- | --- | --- | --- |
|  | β (95% CI) | p |  | β (95% CI) | p |
| Fasting glucose (mmol/L) | 3.2 (0.2, 6.3) | **0.03** |  | 2.5 (-0.9, 5.9) | 0.1 |
| LDL cholesterol (mmol/L) | -3.6 (-5.5, -1.8) | **<0.001** |  | -3.1 (-5.1, -1.1) | **0.003** |
| South Asian ethnicity | 5.8 (2.4, 9.2) | **0.001** |  | 0.9 (-4.9, 6.8) | 0.7 |

**Table 4c.** The associations with maternal ethnic origin, LDL-cholesterol and 2-hour plasma glucose (mmol/L) at 28 weeks' gestation.

|  | Univariate |  |  | Adjusted^1^ |  |
| --- | --- | --- | --- | --- | --- |
|  | β (95% CI) | p |  | β (95% CI) | p |
| 2-hour glucose (mmol/L) | 1.2 (-0.1, 2.5) | 0.06 |  | 0.7 (-0.7, 2.0) | 0.3 |
| LDL cholesterol (mmol/L) | -3.6 (-5.5, -1.8) | **<0.001** |  | -2.8 (-4.9, -0.6) | **0.01** |
| South Asian ethnicity | 5.8 (2.4, 9.2) | **0.001** |  | 1.1 (-4.8, 7.0) | 0.7 |

^1^ Additionally adjusted for age, height, early life socioeconomic position, parity, total fat mass, vitamin B12, folate and 25OH Vitamin D
