# Supplementary figures and images for: Maternal Glucose and LDL-Cholesterol Levels Are Related to Placental Leptin Gene Methylation, and, Together With Nutritional Factors, Largely Explain a Higher Methylation Level Among Ethnic South Asians
Source: Front Endocrinol (Lausanne). 2021 Dec 24;12:809916. doi: 10.3389/fendo.2021.809916 (PMC8739998; doi:10.3389/fendo.2021.809916)

**Figure S1.** Flow chart showing pregnancies selected for analysis.

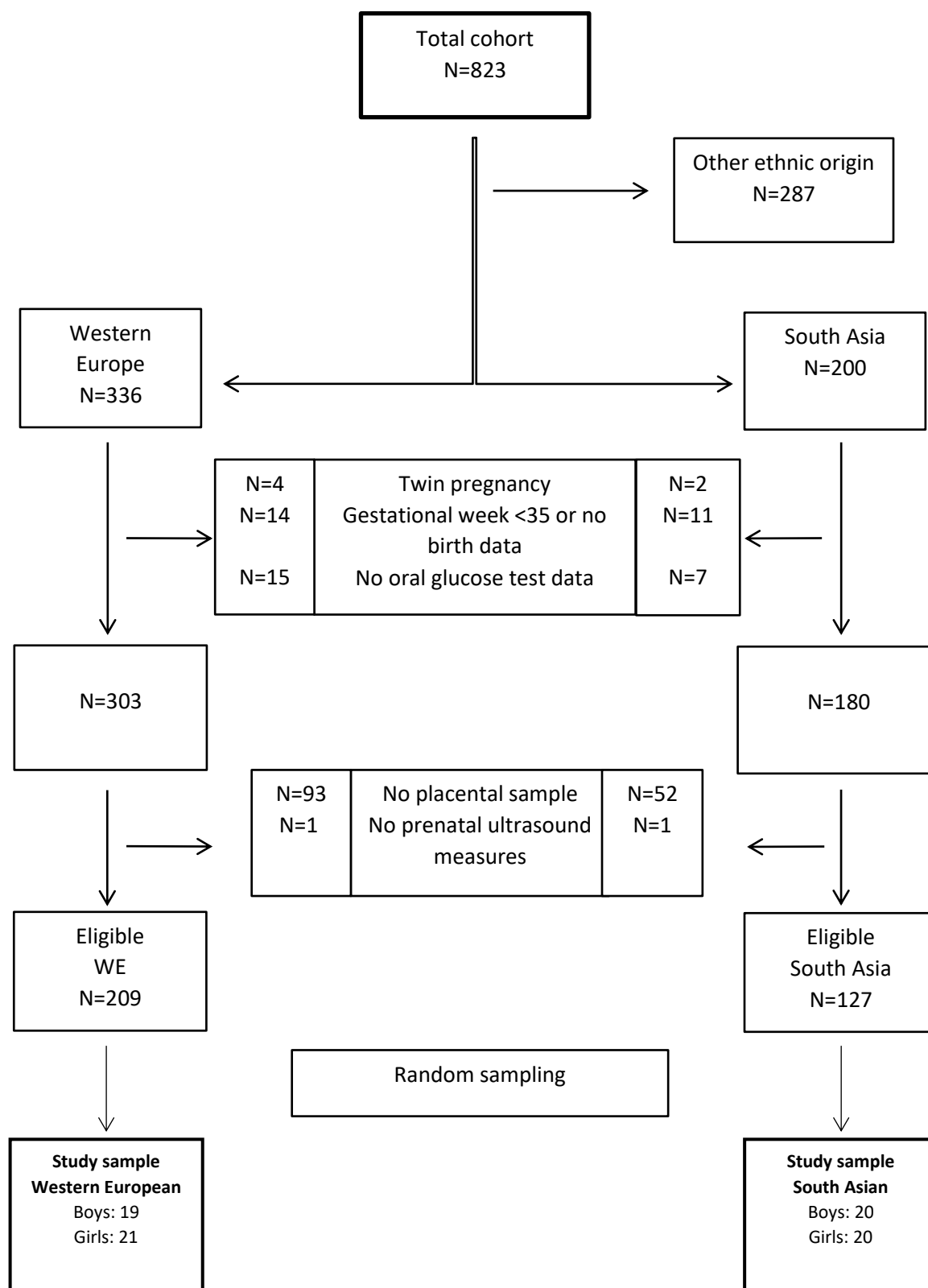

Supplement: Supplementary file 1 [file DataSheet_1.zip › Figure S1 - flow chart.PDF]
